# Regeneration of esophagus using a scaffold-free biomimetic structure created with bio-three-dimensional printing

**DOI:** 10.1371/journal.pone.0211339

**Published:** 2019-03-08

**Authors:** Yosuke Takeoka, Keitaro Matsumoto, Daisuke Taniguchi, Tomoshi Tsuchiya, Ryusuke Machino, Masaaki Moriyama, Shosaburo Oyama, Tomoyuki Tetsuo, Yasuaki Taura, Katsunori Takagi, Takuya Yoshida, Abdelmotagaly Elgalad, Naoto Matsuo, Masaki Kunizaki, Shuichi Tobinaga, Takashi Nonaka, Shigekazu Hidaka, Naoya Yamasaki, Koichi Nakayama, Takeshi Nagayasu

**Affiliations:** 1 Department of Surgical Oncology, Nagasaki University Graduate School of Biomedical Sciences, Nagasaki, Japan; 2 Medical-Engineering Hybrid Professional Development Program, Nagasaki University, Nagasaki,Japan; 3 Biomedical Engineering Course, Advanced Technology Fusion, Graduate School of Science and Engineering, Saga University, Saga, Japan; University of Minnesota Medical Center, UNITED STATES

## Abstract

Various strategies have been attempted to replace esophageal defects with natural or artificial substitutes using tissue engineering. However, these methods have not yet reached clinical application because of the high risks related to their immunogenicity or insufficient biocompatibility. In this study, we developed a scaffold-free structure with a mixture of cell types using bio-three-dimensional (3D) printing technology and assessed its characteristics *in vitro* and *in vivo* after transplantation into rats. Normal human dermal fibroblasts, human esophageal smooth muscle cells, human bone marrow-derived mesenchymal stem cells, and human umbilical vein endothelial cells were purchased and used as a cell source. After the preparation of multicellular spheroids, esophageal-like tube structures were prepared by bio-3D printing. The structures were matured in a bioreactor and transplanted into 10-12-week-old F344 male rats as esophageal grafts under general anesthesia. Mechanical and histochemical assessment of the structures were performed. Among 4 types of structures evaluated, those with the larger proportion of mesenchymal stem cells tended to show greater strength and expansion on mechanical testing and highly expressed α-smooth muscle actin and vascular endothelial growth factor on immunohistochemistry. Therefore, the structure with the larger proportion of mesenchymal stem cells was selected for transplantation. The scaffold-free structures had sufficient strength for transplantation between the esophagus and stomach using silicon stents. The structures were maintained *in vivo* for 30 days after transplantation. Smooth muscle cells were maintained, and flat epithelium extended and covered the inner surface of the lumen. Food had also passed through the structure. These results suggested that the esophagus-like scaffold-free tubular structures created using bio-3D printing could hold promise as a substitute for the repair of esophageal defects.

## Introduction

Esophagectomy is a treatment for conditions including long-gap esophageal atresia, caustic esophagus stricture, and esophageal cancer. In this situation, reconstruction of the esophagus using portions of the more distal gastrointestinal tract, such as the stomach, colon, or intestine may be necessary [1]. However, high complication and mortality rates related to these esophageal reconstructions should be addressed. Problems related to functions of the gastrointestinal tract such as reflux, delayed esophageal transplantation, or dumping syndrome should also be addressed [2, 3]. Moreover, strictures or distensions occurring in the long term require intermittent anastomotic dilatations or re-operations [4].

Tissue engineering is the application of biological, chemical, and engineering principles toward the repair, restoration, or regeneration of living tissues using biomaterials, cells, and growth factors alone or in combination [3]. The three major approaches include guided tissue regeneration using engineered matrices alone, injection of allogenic or xenogenic cells alone, or use of cells placed on or within matrices [5]. The purpose of tissue engineering in the field of treatment of esophageal injury is to strengthen the repair of the injured site or to create a construct to replace the esophagus in case of missing tissues [6, 7]. Recently, some researchers have attempted to create an esophageal substitute by using a natural scaffold approach, while others have investigated artificial prostheses. Those experiments achieved some success, but their results were inconsistent [3, 6–9]. The engineering of an esophageal substitute remains a challenge because of structural characteristics, biocompatibility, and immunogenicity [6, 10].

A technique whereby individual cells adhere to each other and form a spheroid has been considered a promising approach [11]. Nakayama et al. developed a system that makes it possible to construct a tubular structure using only spheroids that contain various cells at an arbitrary rate—the so-called bio-3D printing system [12]. To date, studies have reported using this technique to create blood vessels [13], peripheral nerves [14], cartilage [15], and trachea [16]. Consistent with their methods, we constructed a structure composed of only cells to replace a part of the esophagus and transplanted it into rats. This study aimed to describe the biological features and changes of the esophagus-like scaffold-free structures created using bio-3D printing.

## Materials and methods

### Preparation of multicellular spheroids

Normal human dermal fibroblasts (NHDFs) (Cat No. CC-2511), human umbilical vein endothelial cells (HUVECs) (Cat No. CC-2517), human bone marrow derived mesenchymal stem cells (MSCs) (Cat No. PT-2501), and human esophagus smooth muscle cells (HESMCs) (Cat No. 2710) were purchased. NHDFs, HUVECs, and MSCs were purchased from Lonza, Inc. (Walkersville, MD, USA), and HSMCs were purchased from Sciencell Research Laboratories Inc. (Carlsbad, California, USA). NHDFs were grown in FGM2 medium with the following growth supplements: human fibroblast growth factor-beta (hFGF-B), insulin, fetal bovine serum (FBS), and gentamicin/ amphotericin-B (FGM2 Bullet Kit Lonza, Inc.). HUVECs were grown in EGM-2 containing the following growth supplements: human epidermal growth factor, vascular endothelial growth factor (VEGF), R3-insulin-like growth factor-1, ascorbic acid, hydrocortisone, hFGF-B, heparin, FBS, and gentamicin/amphotericin-B (EGM-2Bullet Kit Lonza, Inc.). MSCs were grown in mesenchymal stem cell growth medium (Lonza, Inc.). HESMCs were grown in smooth muscle cell medium (ScienCell Research Laboratories, Inc.) containing FBS and smooth muscle cell growth supplement. All media were supplemented with 100 U/mL penicillin streptomycin (Gibco-BRL, Palo Alto, CA). All cell lines were cultured on 150-mm tissue culture dishes (TPP, Trasadingen, Switzerland) and maintained in a humidified cell culture incubator maintained at 37°C and 5% CO_2_. The cells were used within 3–9 passages in this study. Structures were fabricated by using a bio-3D printer (Regenova, Cyfuse, Tokyo, Japan) as described by Ito et al. [13]. The cells were detached and collected in 0.25% trypsin EDTA (Nacalai Tesque, Kyoto, Japan) and then centrifuged and re-suspended in the appropriate cell type-specific media.

Different types of structures were made with different cell mixing ratio to confirm the best cell mixing combination for transplantation. Four types of structures were prepared in this study: DF 50%, SMC 50% (group 1); DF 50%, SMC 20%, MSC 30% (group 2); DF 50%, SMC 20%, MSC 15%, HUVEC 15% (group 3); and DF 50%, SMC 20%, HUVEC 30% (group 4) (Table 1). The cell suspension was adjusted to a concentration of 2×10^6^ cells/mL. The mixed suspension was plated onto each well of ultra-low-attachment round 96-well plates (Sumilon PrimeSurface, Sumitomo Bakelite, Tokyo, Japan) at a volume of 100 μl/well and then incubated at 37°C in a humidified atmosphere containing 5% CO_2_. After 72 hours, cells aggregated to form spheroids.

**Table 1:** Cell compositions of spheroids.

	NHDFs	HESMCs	MSCs	HUVECs
**Group 1**	50%	50%	none	none
**Group 2**	50%	20%	30%	none
**Group 3**	50%	20%	15%	15%
**Group 4**	50%	20%	none	30%

NHDFs, normal human dermal fibroblasts; HESMCs, human esophagus smooth muscle cells; MSCs, human bone marrow-derived mesenchymal stem cells; HUVECs, human umbilical vein endothelial cells

#### Bio-3D printing and maturation of tubular structure

**Fig 1:**
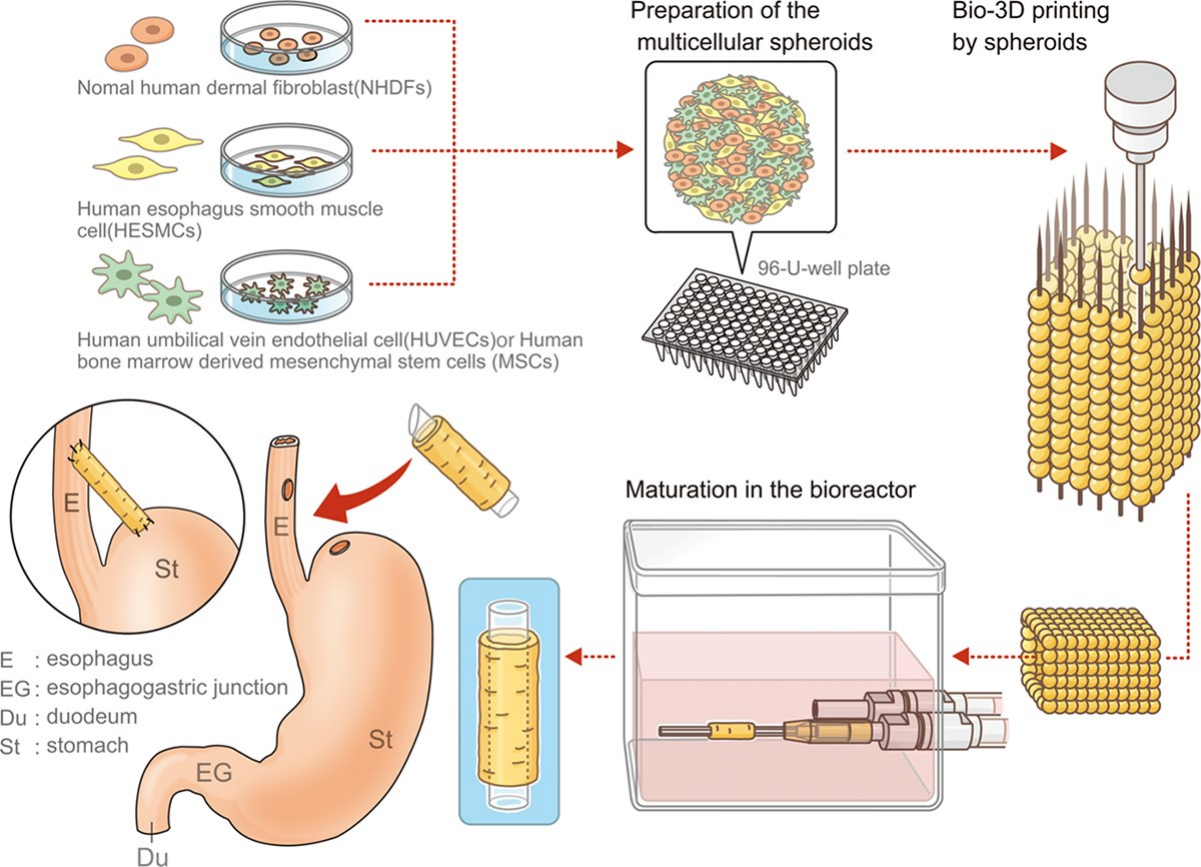
Study overview. Cells: fibroblasts, mesenchymal stem cells, smooth muscle cells, or endothelial cells were cultured respectively. Multicellular spheroids were created using mixed cell suspensions, and the artificial esophagus was then constructed with bio-3D printing using those spheroids. The artificial esophagus was matured in a bioreactor for a total of 4 weeks. Finally, esophageal transplantation of the artificial esophagus was performed.

We used a Regenova bio-3D printer with the Kenzan method (Cyfuse Biomedical K.K. Japan). With the bio-3D printing system, spheroids were robotically harvested one by one and arranged into skewers of a 9 × 9 needle arrays using the 3D-bio printer according to a pre-designed image of a 16-level structure. Following 3D-bio printing, the printed structures were incubated with a proper flow of appropriate medium (200 mL/h) in a bio reactor at 37°C in a humidified atmosphere containing 5% CO_2_ for 1 week. During that week, the spheroids gradually fused to each other and became able to sustain the structure by themselves. Then, the needle array was removed, and the structure was transferred to an intravenous 16-G catheter (Terumo, Tokyo, Japan) and cultured for an additional 3 weeks to promote spheroid self-organization and increase the strength of the structure (Fig 1). The maturation period was set based on the results of our preliminary experiment.

### Morphological and mechanical assessment

At 4 weeks after the structures were bio-3D printed, morphological characteristics of the structures and native esophagus (NE) of rats were analyzed using a machine vision system (Gellex International, Tokyo, Japan). To measure the maximum load tensile extension and tensile strength, their uniaxial tension was tested by a Tissue Puller (DMT, Ann Arbor, USA). Briefly, two small stainless-steel pins were inserted into the inner lumen of the structure as grips. Structures were pulled at a rate of 50 μm/s until their failure. The maximum load tensile extension and tensile strength were calculated.

### Histology and immunohistochemistry

After overnight fixation in 10% formalin (Japan Tanner Corporation, Osaka, Japan), structures were embedded in paraffin and sectioned at 5 μm thickness. They were stained with hematoxylin and eosin (HE) for general histologic evaluation. Immunohistochemistry was performed with the following primary antibodies: alpha smooth muscle antibody (αSMA; 1:200; mouse monoclonal; A5228; SIGMA-ALDRICH, St. Louis, MO, USA) for evaluation of the smooth muscle cell distribution, anti-CD31 (1:400; rabbit polyclonal; bs-195R; Bioss, Boston, MA, USA) for evaluation of the endothelial cell distribution, anti-vascular endothelial growth factor (VEGF; 1:200; rabbit polyclonal; sc-152; Santa Cruz, Texas, USA) for evaluation of vasculogenesis, elastin (1:200; rabbit polyclonal; ab21610; Abcam, Cambridge, UK) for evaluation of generation of ECM, anti-pan-cytokeratin (1:200, mouse monoclonal; ab7753; Abcam) for evaluation of epithelialization, and anti-HLA class I ABC antibody (1:20, mouse monoclonal; ab70328; Abcam) for evaluation of distribution of implanted structure made of human cells. HLA class I ABC is expressed specifically in human cells.

### Animals

Five male F344 rats (10–12 weeks old, weighing 180–230 g) were used in this study. The rats were observed until 30 days after transplantation and then euthanized for analysis. This study was carried out in strict accordance with the recommendations in the Guide for the Care and Use of Laboratory Animals of the National Institutes of Health. The study protocol was approved by the Institutional Animal Care and Use Committee of Nagasaki University (approval number 1607151324).

### Surgery and postoperative management

We selected the structure composed of 50% NHDFs, 20% SMCs, and 30% MSCs (group 2) from among the four types and implemented transplant tests to examine how the structure changed *in vivo*.

Animals were anesthetized by intraperitoneal injection of ketamine at a dose of 100 mg/kg body weight and then intubated. Anesthesia was maintained by inhaled isoflurane and oxygen under intubation. Applying sterile surgical techniques, a left para-rectus laparotomy was made, and the abdominal esophagus segment was exposed. A 2-mm incision was made at the left side of the esophagus with a 1-cm distance from the gastric cardia. A 1.5-cm silicon stent with one side cut at an angle of 45° was inserted into the structure. First, the sharp edge of the stent was sutured to the oral side of the cut esophagus with an 8–0 polypropylene interrupted suture. Then, the structure was sutured to the edge of the cut esophagus with an 8–0 polypropylene continuous suture. Subsequently, the other side of the stent was inserted into the stomach and sutured to it with 8–0 polypropylene interrupted sutures. Following the anastomosis procedure, the transplanted site was covered by the omentum with an 8–0 polypropylene interrupted suture. The laparotomy incision was closed with a 4–0 silk continuous suture in two layers.

All rats were injected with FK506 (0.5 mg/kg/24 h) beginning immediately after surgery to suppress the immune response. The rats were allowed access to food and water ad libitum and were weighed daily under inhaled isoflurane anesthesia during the observation period.

### Statistical analysis

Data are reported as means ± standard deviations (SDs). All statistical analyses were performed using JMP Pro software (version 11.2.0; SAS Institute, Inc., Cary, NC, USA). Comparisons were performed using analysis of variance with Tukey’s honestly significant difference test. Results with P values of less than 0.05 were considered statistically significant.

## Results

### Morphological and mechanical analysis

The mean thickness of the structures was 0.64±0.05 mm in group 1, 0.63± 0.24 mm in group 2, 0.8±0.09 mm in group 3, 0.63±0.18 mm in group 4, and 0.59±0.26 in NE. Although there is no significant difference, the structures in group 3 were thicker than those in other groups (Table 2).

**Table 2:** Results of the tensile test in the bio-3D printed structures and the native esophagus of rats.

	Thickness (mm)	Maximum load tensile elongation (mm)	Tensile strength (N)
**Group 1 (n = 3)**	0.64±0.05	1.92±0.18	0.29±0.081*
**Group 2 (n = 3)**	0.63±0.24	2.11±0.34	0.31±0.011*
**Group 3 (n = 3)**	0.8±0.09	1.24±0.62	0.19±0.011
**Group 4 (n = 3)**	0.63±0.18	2.02±0.27	0.12±0.043
**NE (n = 3)**	0.59±0.26	5.27±0.43	0.46±0.068

Values are expressed as the mean ± standard deviation

* The structures in groups 1 and 2 exhibited significantly greater tensile strength than those in group 4 (P<0.05)

NE; native esophagus

**Fig 2:**
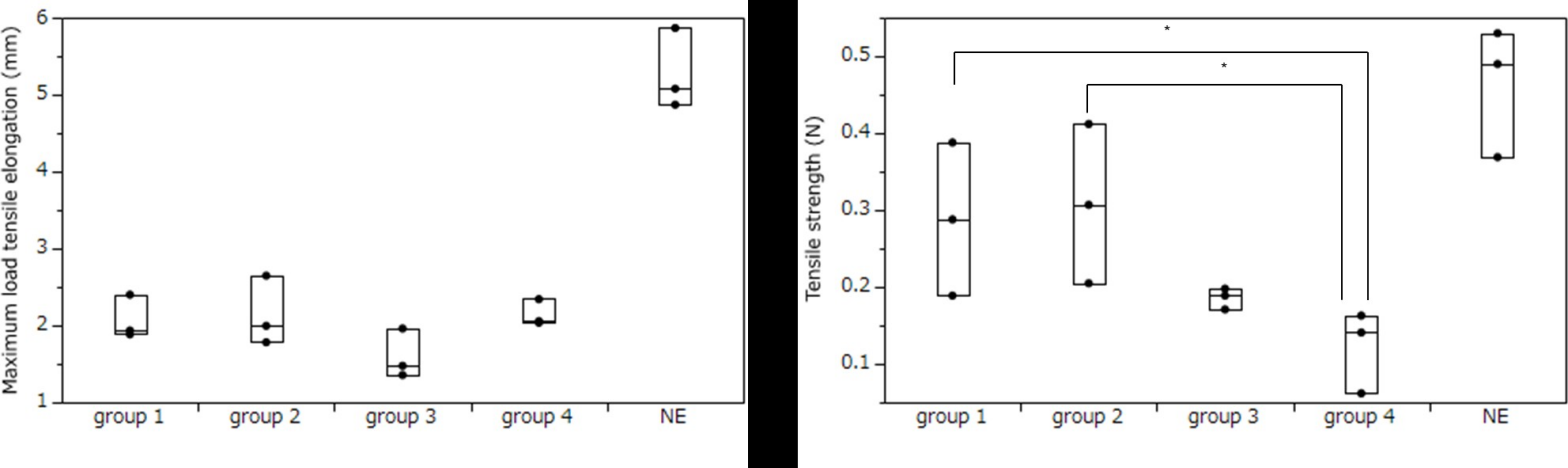
Mechanical characteristics of structures made by the bio-3D printer. A. Maximum load tensile elongation B. Tensile strength The mechanical data of the structures measured by a Tissue Puller.

The mean maximum load tensile elongation was 1.92±0.18 mm in group 1, 2.11±0.34 mm in group 2, 1.24±0.62 mm in group 3, 2.02±0.27 mm in group 4, and 5.27±0.43 in NE (Table 2, Fig 2A). Although there was no significant difference, the structures in group 2 extended longer than those in other groups.

The mean tensile strength of the structures was 0.29±0.081 N in group 1, 0.31±0.011 N in group 2, 0.19±0.011 N in group 3, 0.12±0.043 N in group 4, and 0.46±0.068 N in NE (Table 2, Fig 2B). The structures in groups 1 and 2 exhibited significantly greater tensile strength than those in group 4 (P<0.05). Although there is no significant difference, group 2 exhibited higher tensile strength than the other groups.

Group 2 had the smallest difference in the mean values of maximum load tensile elongation and tensile strength in comparison between structures and NE. Regarding thickness, the diffrence was similar in group 1, group 2, and group 4.

### Histology and immunohistochemistry of the structures *in vitro*

**Fig 3:**
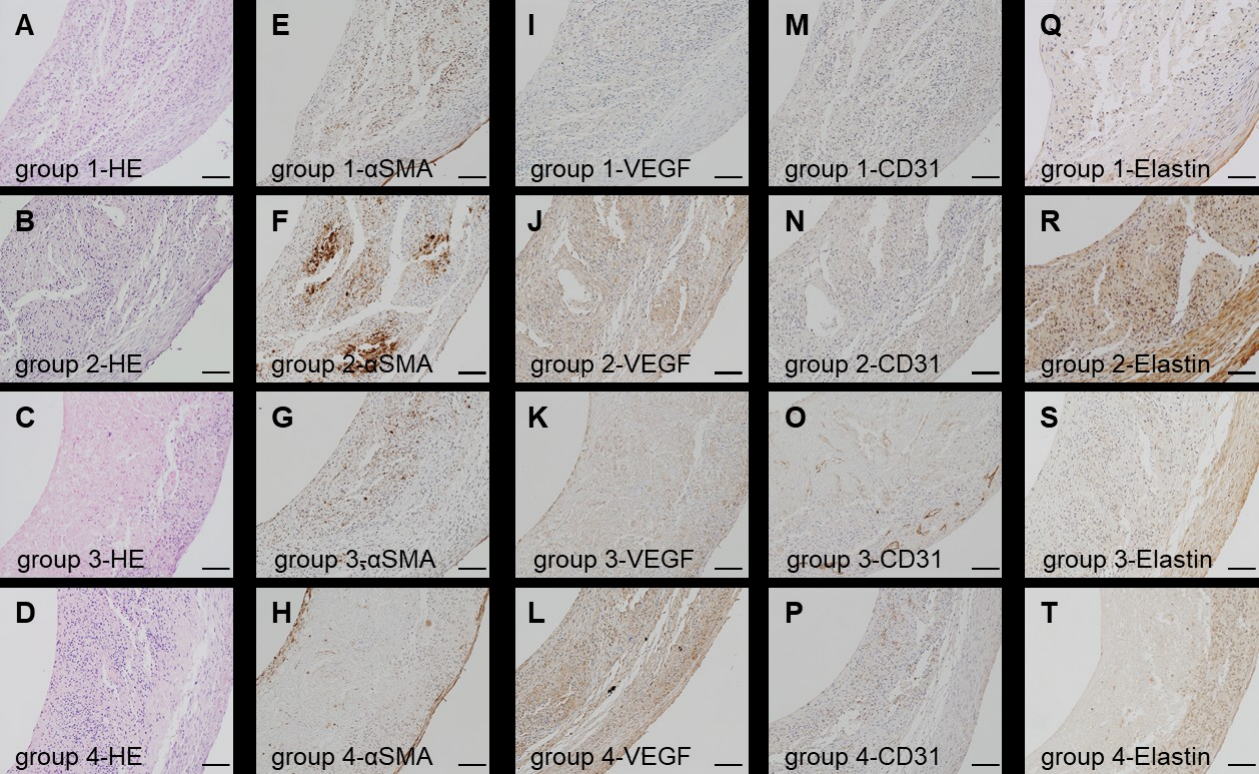
Macroscopic and immunohistochemical findings of pre-transplanted structures. The bio-3D printed structures are shown before transplantation. A-D: HE staining. E-H: Immunohistochemical staining with anti-αSMA antibodies. I-L: Immunohistochemical staining with anti-VEGF antibodies. M-P: immuno-histochemical staining with anti-CD31 antibodies. Q-T: Immunohistochemical staining with anti-elastin antibodies. Scale bar = 100 μm. HE: hematoxylin-eosin, SMA: smooth muscle actin, VEGF: vascular endothelial growth factor.

αSMA-positive cells and VEGF-positive cells were mainly observed in group 2 compared to the other groups. CD31-positive cells were found in group 3 and group 4. In group 3, some micro-vessels were present, consisting of CD31-positive cells. Elastin was expressed mainly in group 2 (Fig 3).

### Postoperative course and macroscopic assessment after esophageal transplantation

**Fig 4:**
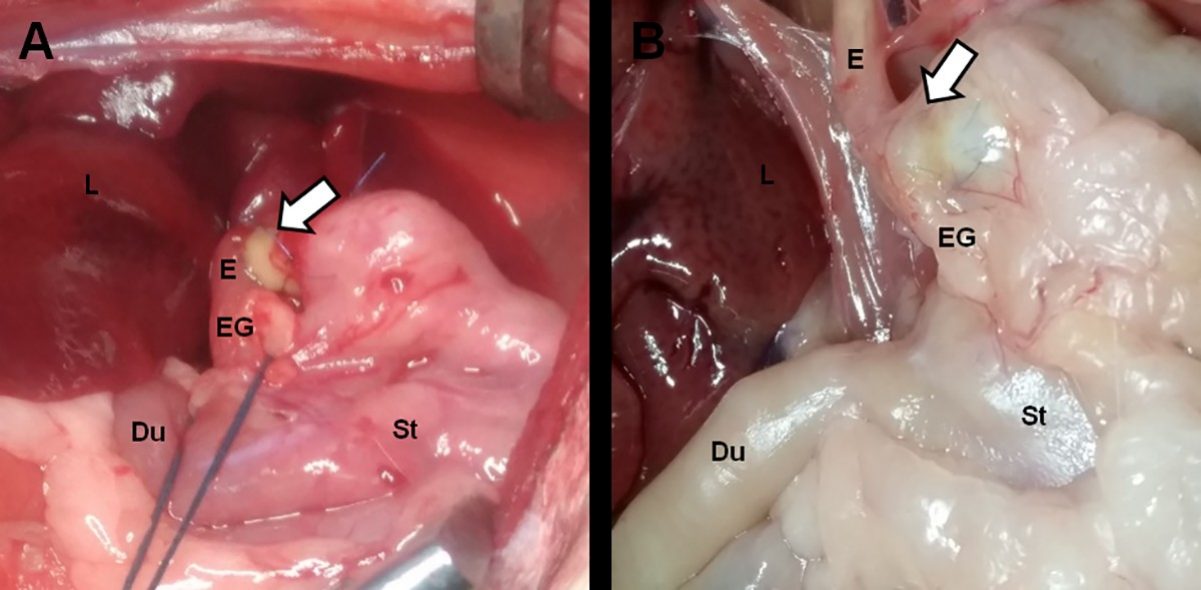
Transplantation of the structures. A. The surgical site of transplantation. B. The transplanted site at 30 days after transplantation. Arrows: transplanted site, Du: duodenum, E: esophagus, EG: esophagogastric junction, L: liver, St: stomach.

**Fig 5:**
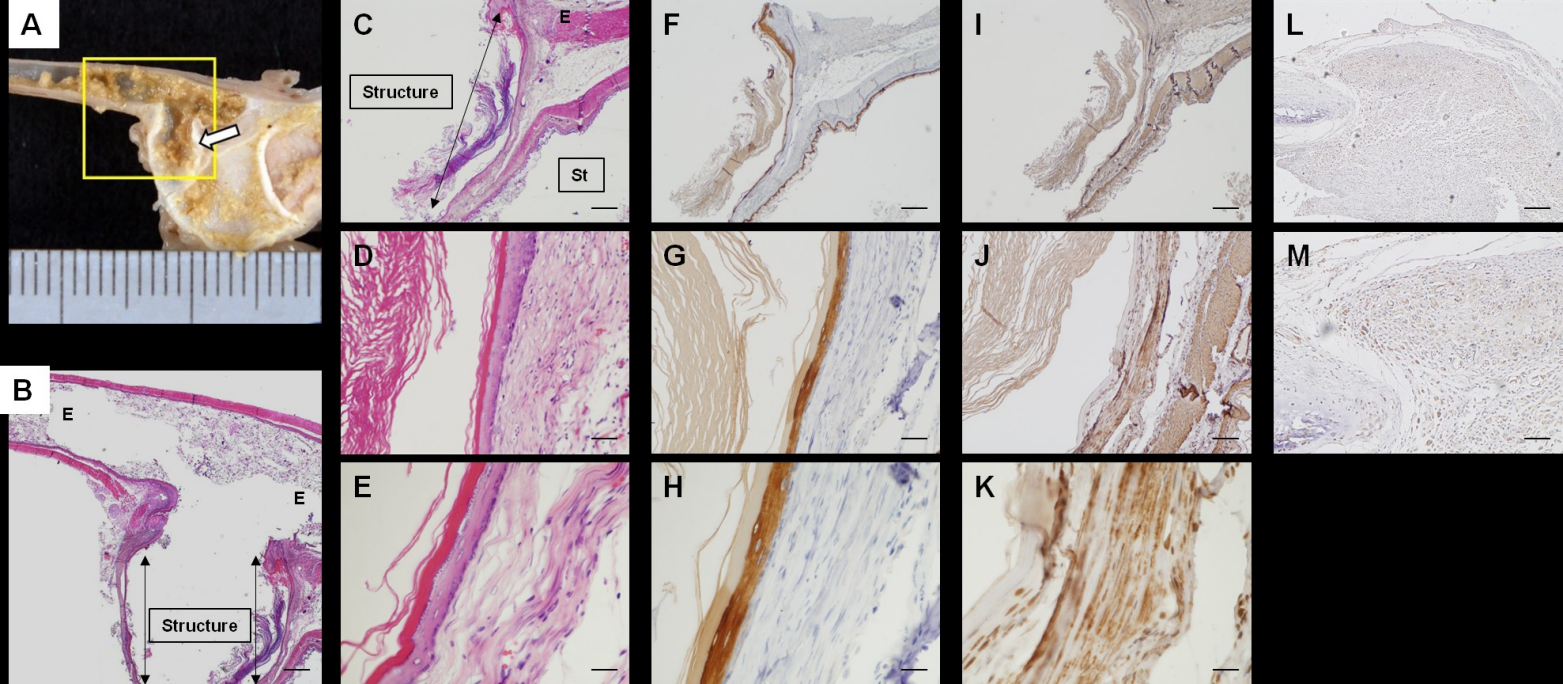
Assessment of the structures at 30 days after transplantation. A: The transplanted site. B: Hematoxylin and eosin (HE) staining at the site shown in A (yellow box). Scale bar = 500 μm. C-E: HE staining of the transplanted structure. Scale bar = 500 μm (C), 100 μm (D), and 50 μm (E). F-H: Immunohistochemical staining of the transplanted structure with anti-pan-cytokeratin. Scale bar = 500 μm (F), 100 μm (G), and 50 μm (H). I-K: Immunohistochemical staining of transplanted structure with anti-αSMA. Scale bar = 500 μm (I), 100 μm(J), and 50 μm (K). Arrows: transplanted structure, E: esophagus, St: stomach. Immunohistochemical staining of the transplanted structure with anti-HLA class 1 ABC. Scale bar = 200 μm (L), 100 μm (M).

All animals survived, and none developed complications after the operation. Minor weight loss was observed during the first postoperative week, but all rats exceeded their preoperative weight by the end of the observation period. After sacrifice and resection of the transplanted esophagus, the transplanted structures had maintained their shape, and there was no leakage or perforation (Fig 4A and 4B). Inside the esophageal structure, food residuals were apparent (Fig 5A). Food residuals implied that the inner lumen of the structure was exposed to gastric juice, but the structure was kept despite their stimulation.

## Histological findings of transplanted structures

At 30 days after transplantation, the boundary between the structure and the esophagus could not be clearly detected on HE staining, as the structures were completely engrafted. The esophageal mucosal epithelium extended into the lumen of the structure and covered its inner surface completely (Fig 5B, 5C, 5D and 5E). Cells on the surface of the lumen expressed pan-cytokeratin (Fig 5F, 5G and 5H). The expression of αSMA was also observed in the structure (Fig 5I, 5J and 5K). Expression of human HLA class I ABC was found only in the part of the transplanted structure, but there were no positive cells in the epithelial layer. This means that the structure made of human cells was maintained in the rats’ body and the native rat epithelium extended on the transplanted structure after transplantation (Fig 5L and 5M).

## Discussion

Although several auto and alloplastic esophageal substitutes have been used for esophageal reconstruction, artificial esophageal replacement remains challenging because of the high incidence of complications such as esophageal stricture [2, 4–7]. Those difficulties mainly arise from the immunogenicity and low biocompatibility of the materials [17]. In recent years, each technology has gradually developed, and good research results have come to emerged [18–21]. Among them, in the report of decellularized esophagus, Guillaume et al. reported that they had transplanted and obtained long-term survival after recellularization of decellularized porcine esophagus, and it is said to be an excellent method in terms of strength and tissue compatibility [21]. Of course, decellularization is an excellent technique, superior in maintaining tissue-specific ECM and high strength, but sacrifice is required to collect ECM. In this respect, the technology we used can make a structure from only cells, which can be a useful method against organ shortage. To overcome these issues, we focused on the novel technology of bio-3D printing and created completely biological, tissue-engineered, scaffold-free structures consisting of multicellular spheroids containing a mixture of cell types via a bio-3D-printer-based system. The present study demonstrates the feasibility of using these structures for esophageal transplantation therapy. We successfully implanted the esophageal tubular structure into rats and found that it was well engrafted with its inner luminal surface fully covered by epithelial cells.

The esophagus mainly consists of cell types including squamous epithelial cells, fibroblasts, and smooth muscle cells, which form the four layers of the esophagus—mucosa, submucosa, muscularis externa, and adventitia [22]. Esophageal substitutes should possess the appropriate mechanical and suture retention strength to withstand the pressure arising from a food bolus [6]. To address this issue, we compared four different structures consisting of different proportions of four cell types. We chose the proportions of 50% NHDFs and 20% HESMCs according to a previous report [13]. Then, we compared the proportion of MSCs and HUVECs to evaluate how they influence the strength and biocompatibility. Endothelial cells play an important role in 3D-printed structures [23–26]; therefore, it is important to include an endothelial cell source. HUVECs can decrease the apoptosis of endothelial cells [24] and form multicellular microvessels in multicellular spheroids [25]. Endothelial cells regulate the activity [27], migration [28], and differentiation [29] of MSCs for the stabilization of newly formed vasculature. Additionally, co-culture of MSCs with SMCs contributed to myogenic phenotype expression of MSCs [30–33], and the use of muscle cells was associated with a decreased inflammatory reaction and enhanced muscle regeneration [4]. To evaluate which combination of cell types was best for artificial esophagus formation and transplantation, the structures were analyzed using tensile test and immunohistochemistry. In the comparison of tensile test between structures, the values of group 2 tended to be larger than those of the other groups in both maximum load tensile elongation and tensile strength. However, it was smaller compared with native esophagus. Although creating a structure with larger maximum load tensile elongation and tensile strength like the native esophagus is a future task in this study, the structure can withstand transplantation using a stent in combination.

In histological findings, the structures in group 2 strongly expressed αSMA and produced elastin. However, there were no CD31-positive cells in the structure, which could produce micro vessels. On the contrary, VEGF expressed at the highest levels in group 2. VEGF is capable of inducing angiogenesis and can boost stem cell-based therapeutic effects [34]. Although microvessel formation might contribute to good engraftment by increasing blood perfusion, we selected the structures of group 2 as the most suitable for transplantation because of their mechanical features and the consideration that VEGF might support the production of microvessels in the structure. The structures of group 2 tended to withstand the greatest force and to demonstrate the largest elongation.

We first attempted the orthotopic procedure for esophageal transplantation in rats. However, the rats did not survive during the observation period because of aspiration pneumonia. Other reports demonstrated the difficulty of replacing esophageal circumferential defects without the use of a silicone stent [35, 36]. Hence, we conducted an interposition procedure with the structure between the esophagus and stomach using a silicone stent to investigate how the structure changed *in vivo*. This procedure was successful, and we were able to demonstrate that the artificial esophagus can contribute to further research into the anatomical repair of diseased esophagus.

Thirty days after transplantation, the structures were found at the transplanted site and contained food residues. We believe that although ingested food mainly proceeded via the host esophagus into the stomach, some boluses with gastric juice moved back and forth between the structure and the stent because there was no esophagogastric junction. Therefore, the structure was exposed to the gastric acid and was tolerant to that stimulation.

One challenge of esophageal tissue engineering is the formation of multilayered structures, because each cell requires a different environment for growth, proliferation, and functional expression [6]. Epithelialization on the surface of the artificial scaffold is considered the most important factor for providing natural esophageal functions in tissue-engineered substitutes as well as protection to the remodeling tissues in the esophageal wall [22, 37]. Epithelial cells have not yet been fully established to show efficacious cell–scaffold interactions or good functionality in artificial organs, thus limiting the success of tissue-engineered grafts [38]. In this study, the lumen of the structure was maintained and covered with cells, all of which were pan-cytokeratin positive. Thus, epithelialization of the luminal surface of the structure was observed even though a stent was inserted. Interestingly, the cells were all stratified squamous cells, which were considered to extend from the esophagus and not from the stomach, even though there was no esophagogastric junction between the structure and stomach. Further investigation is necessary to understand what factors influence epithelialization of the structure. In the outer epithelial layer, smooth muscle cells were maintained, and muscle fiber was apparent. These findings are similar to the anatomy of the native esophagus wall.

This study had several limitations. First, we could not achieve successful results using orthotopic transplantation, as described above, although we could observe how the structure changed *in vivo* using an alternative method. The interposition method employed in this study was useful to confirm the characteristics of the esophageal substitutes created using this new technology in rats. However, it is necessary to achieve orthotopic transplantation as a step forward to clinical application. Second, we did not collect data concerning neurogenesis and peristalsis of the artificial esophagus.

To determine the efficacy of this technology, additional studies with long-term follow-up and removal of the stent after a certain period are needed. The orthotopic transplantation model in large animals will be necessary for clinical applications.

## Conclusion

This study is the first verification for the esophageal structure made with bio-3D printing system. The structures with four different compositions of the cells were made and compared to each other. Although the result was not sufficient compared to the native esophagus, the comparative test suggested that the structure with the highest content of MSCs was shown to be the most suitable for transplantation among the four types of structures. The structure was able to be transplanted and engrafted in the environment exposed to the gastric juice. Even in such a situation, epithelialization occurred in the inner surface of the structure. Although this work is our initial step of the study for the esophageal structure made with bio-3D printing system, it may contribute to the development of treatment for esophageal diseases that require transplantations. The structures created with bio-3D printing technology using a scaffold-free approach showed promise as a potential substitute for esophageal transplantation.

## Supporting information

S1 Table Individual data of the structures and esophagus of rats. (DOCX)
