# Correction: Regeneration of esophagus using a scaffold-free biomimetic structure created with bio-three-dimensional printing

**DOI:** 10.1371/journal.pone.0216174

**Published:** 2019-04-23

**Authors:** Yosuke Takeoka, Keitaro Matsumoto, Daisuke Taniguchi, Tomoshi Tsuchiya, Ryusuke Machino, Masaaki Moriyama, Shosaburo Oyama, Tomoyuki Tetsuo, Yasuaki Taura, Katsunori Takagi, Takuya Yoshida, Abdelmotagaly Elgalad, Naoto Matsuo, Masaki Kunizaki, Shuichi Tobinaga, Takashi Nonaka, Shigekazu Hidaka, Naoya Yamasaki, Koichi Nakayama, Takeshi Nagayasu

There is an error in affiliation 3 for author Koichi Nakayama. The correct affiliation 3 is: Department of Regenerative Medicine and Biomedical Engineering, Faculty of Medicine, Saga University, Saga, Japan.

## References

[1] Takeoka Y, Matsumoto K, Taniguchi D, Tsuchiya T, Machino R, Moriyama M, et al (2019) Regeneration of esophagus using a scaffold-free biomimetic structure created with bio-three-dimensional printing. PLoS ONE 14(3): e0211339 10.1371/journal.pone.0211339 30849123 PMC6408002

