# Supplementary material for: Regeneration of esophagus using a scaffold-free biomimetic structure created with bio-three-dimensional printing
Source: PLoS One. 2019 Mar 8;14(3):e0211339. doi: 10.1371/journal.pone.0211339 (PMC6408002; doi:10.1371/journal.pone.0211339)
Supplement: S1 Table — (DOCX) [file pone.0211339.s001.docx]

|  | Tensile strength (N) | Thickness (mm) | Maximum load tensile elongation (mm) |
| --- | --- | --- | --- |
| group 1 | 0.19 | 0.62 | 1.94 |
| group 1 | 0.29 | 0.69 | 1.89 |
| group 1 | 0.39 | 0.63 | 2.4 |
| group 2 | 0.41 | 0.48 | 1.78 |
| group 2 | 0.21 | 0.62 | 2.65 |
| group 2 | 0.31 | 0.79 | 2 |
| group 3 | 0.19 | 0.89 | 1.36 |
| group 3 | 0.19 | 0.79 | 1.48 |
| group 3 | 0.17 | 0.74 | 1.96 |
| group 4 | 0.16 | 0.55 | 2.34 |
| group 4 | 0.14 | 0.76 | 2.34 |
| group 4 | 0.62 | 0.58 | 2.06 |
| NE | 0.37 | 0.63 | 5.08 |
| NE | 0.49 | 0.57 | 5.87 |
| NE | 0.53 | 0.58 | 4.87 |

S1. The individual data of the structures and the esophagus of rats.
